# Hormonal computing: a conceptual approach

**DOI:** 10.3389/fchem.2023.1232949

**Published:** 2023-08-16

**Authors:** Jordi Vallverdú, Max Talanov, Alexey Leukhin, Elsa Fatykhova, Victor Erokhin

**Affiliations:** ^1^ ICREA Academia, Universitat Autònoma de Barcelona, Bellaterra, Spain; ^2^ Institute for Artificial Intelligence R&D, Novi Sad, Serbia; ^3^ Laboratory of Neuromorphic Computing and Neurosimulatons, Kazan Federal University, Kazan, Russia; ^4^ B-Rain Labs LLC, Kazan, Russi; ^5^ Children’s Republican Clinical Hospital, Ministry of Health of the Republic of Tatarstan, Kazan, Russia; ^6^ Istituto dei Materiali per l’Elettronica ed il Magnetismo, National Research Council (CNR), Parma, Italy

**Keywords:** hormonal computing, hormones, bioinspiration, signaling, programming, sensors

## Abstract

This paper provides a conceptual roadmap for the use of hormonal bioinspired models in a broad range of AI, neuroengineering, or computational systems. The functional signaling nature of hormones provides an example of a reliable multidimensional information management system that can solve parallel multitasks. Two existing examples of hormonal computing bioinspired possibilities are shortly reviewed, and two novel approaches are introduced, with a special emphasis on what researchers propose as hormonal computing for neurorehabilitation in patients with complete spinal cord injuries. They extend the use of epidural electrical stimulation (EES) by applying sequential stimulations to limbs through prostheses. The prostheses include various limb models and are connected to a neurostimulation bus called the central pattern generator (CPG). The CPG bus utilizes hormonal computing principles to coordinate the stimulation of the spinal cord and muscles.

## 1 Introduction

Bioinspiration has been a fundamental source of ideas for the advancement of computer sciences (Floreano and Mattiussi, 2008). Because of hormones’ fundamental role in living systems, we consider the benefits of addressing their simulation in computer scenarios, which is an approach scarcely explored. Hormones are a fundamental chemical signaling mechanism working in all living systems, from humans (Norman and Henry, 2022) to other mammals (Young et al., 2011), fishes (Liley and Stacey, 1983), insects (Nijhout, 1998), plants (called “phytohormones” (Koepfli et al., 1938), slime mold (Chen, 1975), bacteria (Sperandio et al., 2003), and even viruses (Huang et al., 2019). The endocrine system secretes hormones that coordinate responses to stimuli in a specific slower and longer-acting way. Consider, for example, the growth activation. However, they can also activate quick responses, like the release of epinephrine and the flight-or-fight hormone, or even regulate circadian rhythms (melatonin), among a long list of functions (reproductive functions, blood pressure regulation, heart rate modulation, muscle tone definition, digestion management, etc.).

From an information processing and functional perspective, hormones provide the signaling of essential actions, after some sensory cells detect some event and release hormonal responses targeted to the other cells (Kushiro et al., 2003). A specific hormonal messaging is combined with other decision-making systems (following a multilevel *if then* biochemical command pattern), as we see in animals with a nervous system. The factors that control hormone secretion are diverse: stimulatory and inhibitory agents, other hormones, and external factors (for example, related to the circadian rhythm). Such hormonal changes also affect cognitive processes, such as attention, surprise, and learning, playing a fundamental role in the release of the hormone noradrenaline (Breton-Provencher et al., 2022). They can even affect mammalian brains during specific temporal conditions, like pregnancy, motherhood, and parenthood (Lambert and Kinsley, 2012; Martínez-García et al., 2021).

Hormones can travel throughout the body, but they only affect specific areas that have sensors capable of recognizing and responding to those signals. These sensors, known as target cells, are responsible for converting the external chemical signals of hormones into internal cellular responses. In other words, hormones can communicate with certain parts of the body that are equipped with the necessary receptors to interpret and react to their signals.

In our previous work (Vallverdu, 2022), we explored succinctly possible applications of hormonal model-based systems to computational scenarios. In this paper, we aim to define a roadmap to the multiple options of hormonal computing, which we summarize in four different directions:

1. Bioinspired hormonal computer systems.
2. Bridges between living systems.
3. Programming languages.
4. Hybrid human–machine technologies.

The first direction is related to the design of computational architectures (for example, programs) (Brinkschulte et al., 2008), one example of which is included in a special edited book on organic computing that follows the principles of bioinspiration. The second direction is related to the use of potential unexplored ways to interconnect different living systems thanks to their hormonal signaling interfaces implemented using engineering devices. The third direction implies the creation of programming languages that allow the introduction of programming commands based on hormonal messaging that globally affects the weights of other computing commands. Thus, the release of some global hormonal commands can produce a general impact on the semantics by which programming commands are performed and interpreted at the syntactic level. The fourth direction considers the implementation of interfaces to a body of living systems that are directly related to hormonal communication. Therefore, in Sections 2, 3, we explore already existing and promising paths for bioinspired hormonal computation, while in Sections 4, 5, we provide original research.

## 2 Bioinspired hormonal computer systems

One way to draw inspiration from hormones in the field of computer systems is by developing computer simulations that mimic certain aspects of hormonal systems to manage computer facilities. For example, in Brinkschulte et al. (2008), the use of an artificial hormone system to enable self-organization and real-time task allocation in organic middleware was explored. This approach involves creating computer systems that replicate some of the characteristics and functions of hormones found in living organisms. The authors created the middleware for distributed system orchestration while designing the management infrastructure of computational systems built from a large number of heterogeneous processing elements. The current demand for distributed systems raises the question of novel ways to design and manage them. The authors inspired themselves with organic computing ideas, considering a computational device as a dynamic system that is able to self-organize. The hormonal-inspired middleware as a self-configurable and distributed system autonomously selects an initial task allocation, i.e., finds the best initial processing element for each task. The authors indicate that the term “artificial hormone system” was chosen because their approach was highly inspired by the hormone system of higher mammals, which, in this case, was applied to computational task distribution on heterogeneous processing elements even implanted in the human body.

Following similar ideas, Szkaliczki et al. (2013); (2016) introduced an artificial hormone system capable of handling the dynamics of complex systems, exploiting the existing analogy between the Markov chains and the artificial hormone system. According to their statement, the problem of content placement is considered to be NP-complete, meaning it is computationally difficult to solve. It is also closely connected to other challenging problems such as edge-disjoint path routing, scheduling, and the bin packing problem. In simpler terms, finding an optimal solution for content placement is a complex task with similarities to various other difficult problems in computer science.

**FIGURE 1:**
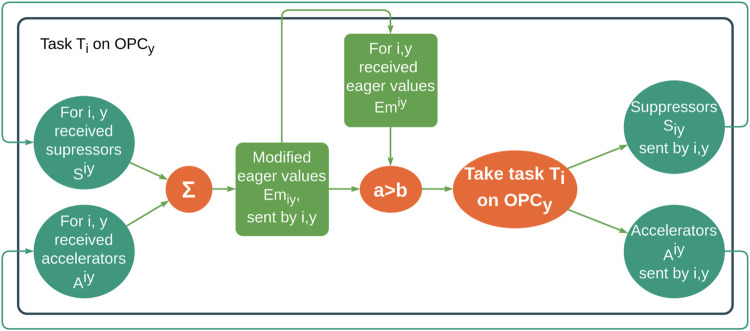
Hormonal loop schematic (Elmenreich et al., 2021), redesigned by the authors, where the notation is *H* ^*iy*^ hormone for task *T*_*i*_ executed on *OPC*_*y*_ and *H*_*iy*_ hormone from task *T*_*i*_ executed on *OPC*_*y*_. Latin letters represent task indices, and Greek letters represent processing element indices.

Some years later, Elmenreich et al. (2021) created their artificial hormone system (AHS), as part of organic middleware for mapping tasks on a heterogeneous grid of processing elements. It works completely decentralized, and AHS cannot be controlled by a single processing element; instead, the hormone values have to be selected carefully to guarantee system stability, defined between upper and lower bounds which have to be met to guarantee system stability. For task allocation, three types of hormones were used: eager value, suppressor, and accelerator. Thus, this hormonal-based control loop allowed the system to be self-configuring, self-organizing, self-optimizable, and self-healing (as shown in Figure 1).

**FIGURE 2:**
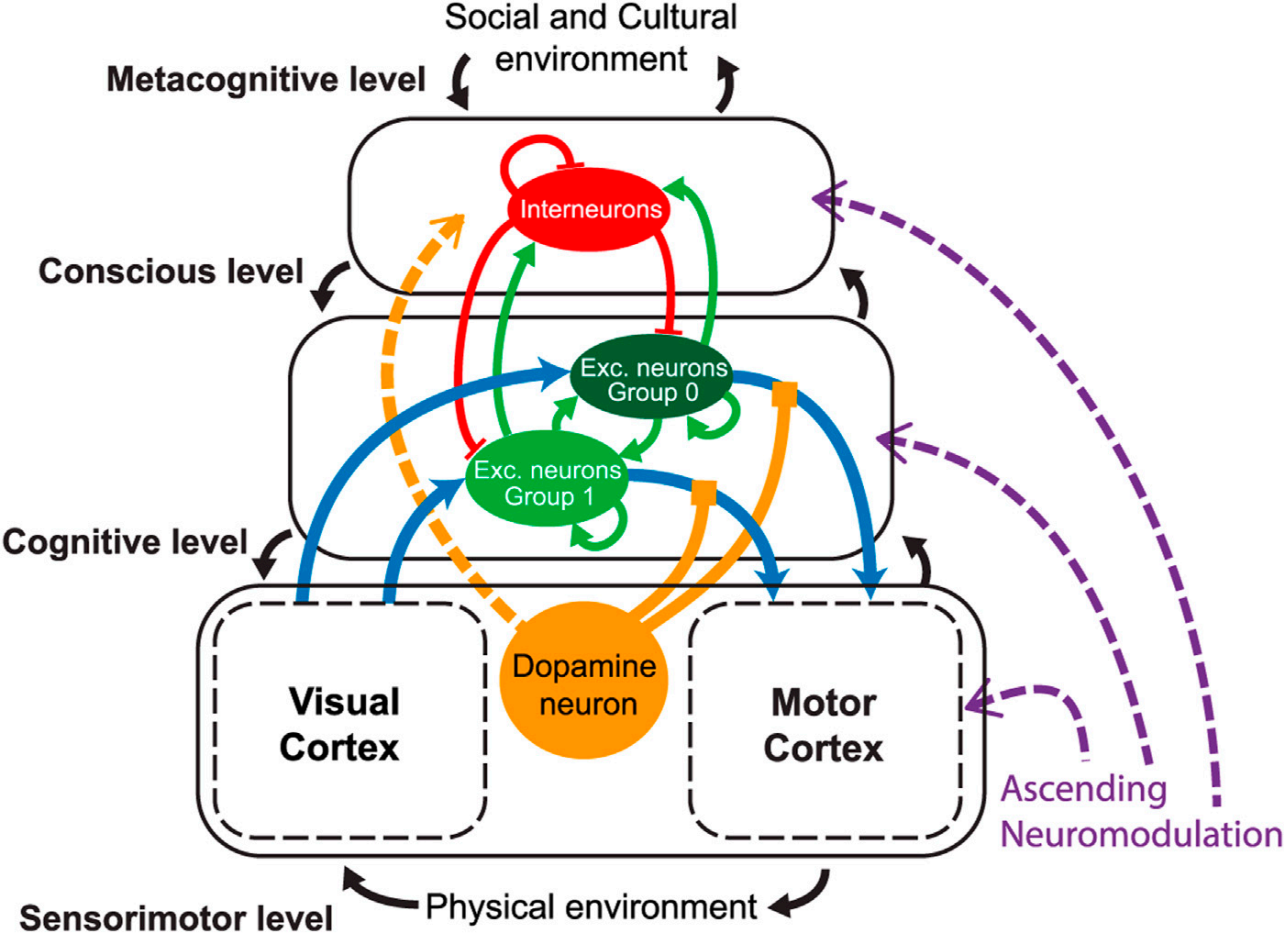
Levels of action in humans according to the hormonal mechanisms. According to Volzhenin et al. (2022), hormonal mechanisms in humans operate at different levels of action. These levels include the hypothalamic–pituitary axis, which regulates the release of hormones from the brain’s hypothalamus and pituitary gland, and the endocrine glands located throughout the body. Hormones released by these glands travel through the bloodstream, reaching various target cells and tissues. At the cellular level, hormones interact with specific receptors on target cells, initiating intracellular signaling pathways that lead to various physiological responses. These responses include changes in gene expression, alterations in cellular metabolism, or modifications in the function of specific organs or systems. Understanding the levels of action in hormonal mechanisms is crucial for comprehending how hormones influence different aspects of human physiology and behavior. By studying these mechanisms, researchers can gain insights into the regulation of bodily functions, the maintenance of homeostasis, and the coordination of various physiological processes (Volzhenin et al., 2022).

On the other hand, exploratory research on neural networks (Volzhenin et al., 2022) explains the emergent properties of cognitive systems using the introduction of the dopamine neurotransmitter, acting in all the existing functional layers. The dopamine appears necessary to properly provide credit assignment despite the temporal delay between perception and reward (Figure 2).

This is exactly the role of hormones: not only to act as triggers for some changes in the body but also as direct or indirect regulators of even high cognitive processes. Thus, the impact of the hormonal system can be understood as transversal to the whole body, underrating itself as an extended and enactive system. For that reason, bioinspired computer hormonal systems are useful for orchestrating complex systems, as we have demonstrated with previous examples.

## 3 Living bridge interfaces

We see hormonal bioinspiration as a very useful strategy in the engineering of interfaces between different living systems. Dehshibi et al. (2021) used the advantage of fungi cells sensing extracellular signals via reception, transduction, and response mechanisms, allowing them to communicate with their host and adapt to their environment. Once shown that *Pleurotus* oyster fungi generate electrical potential impulses in the form of spike events as a response to several causes, they explored how such fungi could act as sensors of human secretions such as hormones. Consequently, they exposed *Pleurotus* oyster fungi to hydrocortisone (a hormone replacement that is similar to the natural stress hormone cortisol) and studied their reactions. Later, the authors demonstrated the causal interaction between fungi and human hormones. They explored possible future adaptive fungal wearables capable of detecting human physiological states and then acting as biological sensors for different engineering solutions (temperature regulation and medical sensors). In addition to this initially cited research, the authors provided supplementary evidence that supports ecological studies (Chiolerio et al., 2022). It must be said that Adamatzky et al. (2021) offered a valuable extension to the discussion, focusing on smart wearables that process information from the user and environment, reporting results as electrical signals. Therefore, fungi show promise for eco-friendly biowearable technologies. They also noticed that experiments with oyster fungi on hemp fabric revealed their sensing potential, inspiring intelligent sensing patches for future fungal wearables.

The European research project *SENSHOR* (Grant Agreement ID: 749973) combines ideas and practices from experts of Boston and Bordeaux universities. It also focuses on a wearable sensor for hormones using native microbial sensing (Grazon et al., 2020). Such biosensors capture a steroid hormone, progesterone. Using proteins named transcription factors, the researchers were able to sense hormones, and their technology makes it feasible to integrate these types of sensors in wearable devices.

## 4 High-level design of the approach

In this section, we focus on design of computational systems with embedded hormonal-like mechanisms. We propose the combination of two different ideas: directed acyclic graphs (henceforth DAG) and weighted logic. DAGs are mathematical structures used to represent relationships or dependencies between objects or events. They are commonly used in various fields, including computer science, mathematics, and graphical models. In the context of graphical models or causal inference, DAGs are used to depict causal relationships between variables, and they can help in the understanding of the qualitative aspects of causal relationships. Analyzing the structure of the DAG, researchers can make qualitative inferences about the presence or absence of causal connections between objects or variables. However, it is noteworthy that DAGs alone do not provide quantitative information about the strength or magnitude of causal relationships. To determine the quantitative aspects of causality, additional statistical methods and data analysis techniques are required. In summary, while DAGs can assist in the understanding of qualitative aspects of causal relationships, they do not inherently define something qualitatively on their own. In our case, DAGs capture and explain the causal and retro-feeding cycles of hormones. Then, it can be easily adapted and translated into computational scenarios.

**FIGURE 3:**
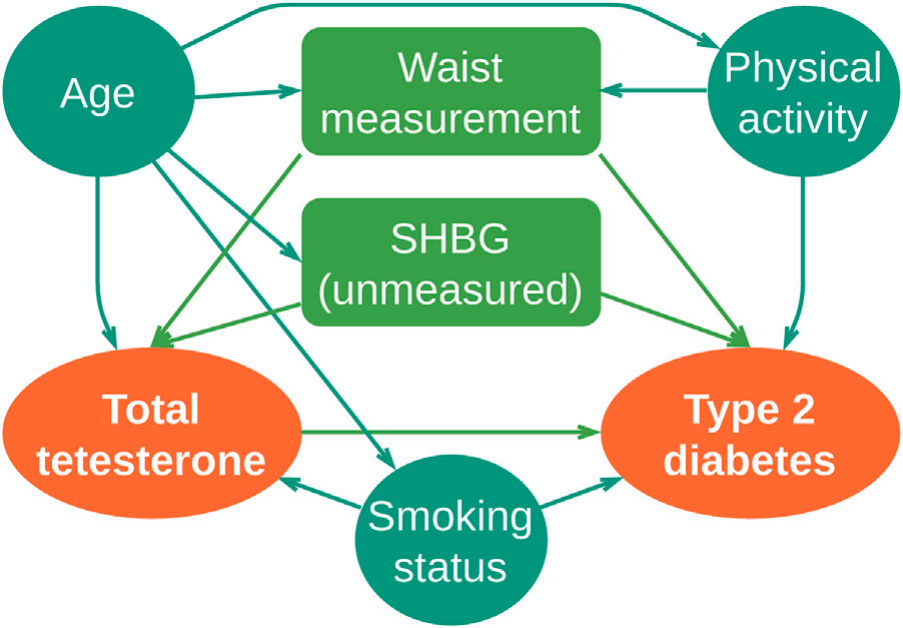
DAG diagram of type 2 diabetes: total serum testosterone at baseline; outcome: incident type 2 diabetes for follow-up; covariates: age, smoking status, waist circumference, and physical activity and sex hormone-binding globulin (SHBG) as an unmeasured variable. Colliders framed: waist measurement and SHBG. Adapted by the authors from Schipf (2011)

DAGs can easily capture the functional causal directions of hormonal processes, as described previously in Schipf (2011) (adapted and translated in Figure 3).

The option to create causal graphs like DAGs provides a reliable mechanism to map, understand, and reproduce/adapt the dynamics generated by hormonal systems. DAGs are capable of performing it and, at the same time, maintaining logical causal connectivity and connection’s transparency (Pearl, 1995; 2009). DAG illustrates the dependencies amongst a set of processes and resources (as we identify both as nodes in the DAG). They are also very useful in catching deadlocks within a functional cycle. Therefore, a DAG provides, at the computational programming level, a topological description of the necessary sequential design of the system. Programming languages use DAG really widely to connect internal representation of objects. It also makes it possible to enhance the performance of the code by either eliminating or rearranging the code lines, as well as critical deadlocks. DAGs are also used in compiler design as a tool that depicts the structure of basic programming blocks, helps to see the flow of values flowing among the basic blocks, and offers optimization. Git, a distributed version control system (Loeliger and McCullough, 2012), uses DAGs as a commit tree pattern (tracking dependencies), with plenty of sub-tasks: content storage, reference pointers for heads, object model representation, and remote protocol.

Adding bioinspiration from the hormonal system into DAGs, we find a new option: to use weighted logic as an integrated way of adding quantitative values (that can allow the conditions of triggers or suppressors) to the commands, creating a hormonal network similar to neural. The functional property of our interest in relation to hormonal bioinspiration is the time-lapse of hormonal messaging and modulations. Hormonal changes inside a body follow a different speed in relation to the central nervous system (CNS): while the CNS uses fast and hardwired signal channels, the hormonal system acts much slower (and permanently) and spreads hormones using slower channels, like bloodstream through vessels (Kolka and Bergman, 2012). The CNS and hormonal systems run in parallel describing multi-levels of actions, which have different speeds and preferences, creating complex dynamic systems.

We propose to use DAGs and weighted logic together to create bioinspired hormonal networks, by (a) defining the nodes (identifying the relevant nodes and axes, taking into account factors that play a role in the system one wants to model, functions of these nodes, different components, processes, or states within the system); (b) establishing causal relationships (using the network to specify the causal relationships between the nodes, where the network represents the flow of influence or dependencies or causality among the nodes); (c) assigning weights to axes (weighted logic)—a weight represents the strength of the causal relationships; (d) propagating signals—one can simulate the computational model of propagating signals through the network; and (e) creating feedback and iterative refinement of parameters during the network/model life cycle. The statistical analysis of the simulated data and comparison with biological data is possible and could be recommended. If necessary, refine the model by adjusting the weights or modifying the structure of the DAG to better capture the dynamics of the hormonal system. By combining DAGs to represent causal relationships and weighted logic to assign strengths to those relationships, computational models can mimic the behavior of the hormonal system. This approach allows for the exploration of complex interactions and emergent properties that arise from the interplay of variables, similar to how hormones regulate various processes in biological systems.

**FIGURE 4:**
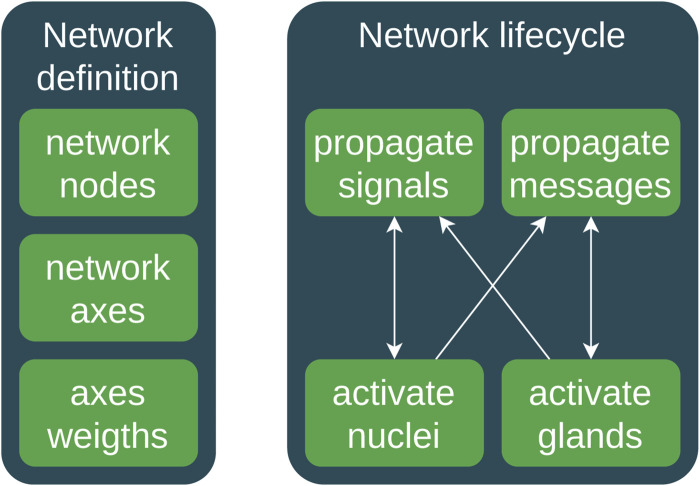
High-level activity diagram of the main messaging and signaling workflow. **(1)** DAG network definition: **(1a)** definition of network nodes; **(1b)** network axes; **(1c)** axes’ weights. **(2)** The life cycle contains the following stages: **(2a)** propagation of the neuronal signals or spikes; **(2b)** triggering and activation of nuclei via signals; **(2c)** propagation of hormonal messaging via bloodstream; **(2d)** activation of hormonal glands.

An example of a pseudocode that demonstrates how to emulate a simplified hormonal process using DAGs and weighted logic is given in Figure 4.

In Figure 4, we present two types of signal/messaging propagation to identify the quick neuronal signal transmission and slow hormonal messaging. There is a connection between neuronal and hormonal systems; thus, we use both neuronal and hormonal networks as integrated neuro-hormonal systems with two signal propagation mechanisms. The *propagate*_*signals* and *propagate*_*messages* functions calculate the weighted sum of inputs for each variable based on the network axes’ weights. They later update the parameters of nuclei and glands by applying an *activation*_*function*, taking into account delays.

This is a simplified example. Considering that real hormonal systems are much more complex, we see that this approach serves as a starting point to demonstrate the concept of using a network combined with DAGs and weights to simulate hormonal processes in a computational model.

The bioinspired computational approach using hormonal mechanisms provides option design computational systems using multilevel ways of interaction, releasing specific responses only under very constrained conditions. Similar to the CNS and hormonal system, hormones cannot reach the high speed and complex role of the CNS, but they generate changes in the whole body and affect the CNS. The use of weighted logic can allow the implementation of DAG models that facilitate dynamic algorithms (Devienne and Lebegue, 1986; Cohen et al., 2008). The weighted way of operating can capture the way by which hormones act in biological systems (bodies): creating a dynamical remapping of the values being computable, activating or silencing signals which activate responses or modular performances.

## 5 Middle-level design of implementation with hybrid technologies

**FIGURE 5:**
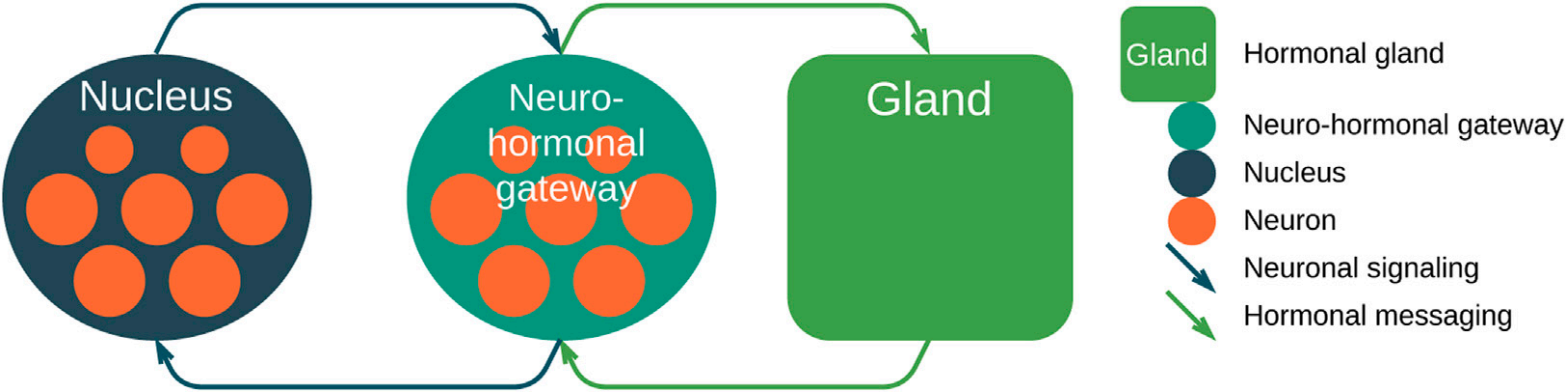
High-level representation of the proposed neuro-hormonal messaging architecture. **(1)** Neurons of the nucleus spiking form the inbound signal of the neuro-hormonal gateway; **(2)** later, the gateway propagates the hormonal message to the gland; **(3)** the gland, in turn, sends feedback to the gateway; and **(4)** the gateway sends feedback to the nucleus. Messaging is performed using the fibers.

The previously described approach for the neuro-hormonal integration using networks and two different propagation activation mechanisms could be used as the backbone of the infrastructure of orchestrated heterogeneous implants (DiLorenzo and Bronzino, 2008). There are two subsystems in the proposed model: (1) the artificial hormone messaging system (Renteln et al., 2011) and (2) the neurosimulation signaling system used for the neuro/bio-interfaces (Talanov et al., 2023; 2021). The main components involved in the relationship between neuro-hormonal computing and messaging/signaling are presented in Figure 5. We use two types of information transfer in the architecture: (1) *neuronal signaling*—fast electrical signal propagation along the fibers or neuronal offshoots and (2) *hormonal messaging*—slow diffusion-based propagation of active agents through the medium (body). For the proposed architecture, we use four types of messages: (1) a *accelerates* b, (2) a *suppresses* b, (3) a *excites* b, and (4) a *inhibits* b, where types (1) and (2) are hormonal messaging, types (3) and (4) are neuronal signaling, and a and b are nodes. We use different terms for similar transport mechanisms to emphasize the distinction and roles in the proposed approach.

Here, (1) the first network group of nodes or nucleus consists of individual neurons that generate signals, and superposition of them forms the nucleus signal that is transmitted via fibers (neuronal signaling via weighted axes, depicted as dark green arrows); (2) the second group of nodes or neuro-hormonal gateway receives the neuronal signal and generates the hormonal messages via, for example, bloodstream (hormonal messaging, depicted as light green arrows) and transmits it to the fourth group of nodes—hormonal gland; (3) the gland, in turn, sends feedback via hormonal messaging transport; and (4) later, the neuro-hormonal gateway sends feedback using neuronal signaling.

### 5.1 Node: nucleus/gland

#### 5.1.1 Nucleus

A nucleus (group of nodes) is a set of neurons (nodes) with the distributions of the following parameters.

1. *Activation function* or *fitness* identifies how good the cell is for the particular hormonal message or neuronal signal processing (it could be understood as the distribution of particular receptor type densities of each cell membrane).
2. The *propagation function* includes:
  a. During the *refractory period*, cells have the distribution of durations, while they are not able to generate sequential spikes if they have generated one previously.
  b. The *processing speed* is influenced by membrane conductance and capacitance.

**FIGURE 6:**
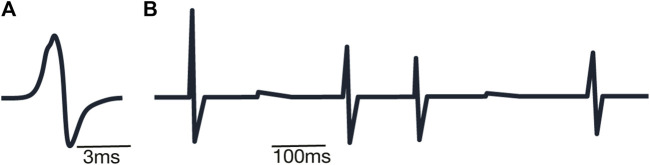
Examples of spike shapes: **(A)** example of simulated extracellular spike shape; **(B)** example of sum of simulated extracellular voltages.

Furthermore, the activation function in case of *fitness* reached the threshold value: this means the node fits. Further processing in the node is performed in the following way: the simplified excitation level as the representation of the membrane potential of a neuron is calculated according to the following formula:

$$L_{\mathrm{neuron}}\left(t\right)=\overset{n}{\underset{i=1}{\sum }}w\left(t\right)+leakage\left(t\right)+noise,$$ (1)

where *L*_*neuron*_ is the abstract excitation level; *w* is a weight or representation of synaptic conductance, positive stands for excitatory and negative for inhibitory synapses; *leakage* is the value corresponding to the leakage current or the speed with which the subthreshold value *L* returns to the resting value; and *noise* is the value of stochastic input. The low computational burden of Eq. 1 is helpful to balance bio-compatibility and computing performance to be used as one of the main components of the bio-compatible infrastructure of neuro-hormonal message bus. To compute the superposition of membrane levels (or local field potential) previously presented as levels, we have to take into account the signal (spike) times, and then, we can sum up the potential at the particular moment in the following way:

$$L_{\mathrm{nucleus}}\left(t\right)=\overset{n}{\underset{i=1}{\sum }}L_{\mathrm{neuron}}\left(t\right),$$ (2)

where *L*_*neuron*_ is calculated according to Eq. 1 and *L*_*nucleus*_ represents the simplified superposition of neuronal levels included in the nucleus to decrease the computational burden Here, for the sake of computational efficiency, we use the sum as the primitive implementation of the superposition of the electrical fields forming local field potential. This way the overall nucleus level curve is created via the sum of individual neuron levels (Figure 6B), taking into account signal (spike) times (Figure 6A).

#### 5.1.2 Gland

A gland, similar to a nucleus, is the representation of the group of nodes, with only one difference: it produces the hormonal messages instead of neuronal signals and thus uses hormonal messaging transport (bloodstream) instead of neuronal fibers.

When a hormonal activation or suppression message reaches a group of nodes, Eq. 3 identifies the levels of the nodes under the influence of hormonal messages (taking into account that hormones influence any node):

$$L_{\mathrm{node}}\left(t\right)=\overset{n}{\underset{i=1}{\sum }}L_{\mathrm{gland}}\left(t-delay\right),$$ (3)

where the *L*_*node*_ is computed similar to the *L*_*neuron*_ from Eq. 1. The level is increased in case of acceleration messages and decreased in case of suppression messages. When it reaches the threshold value, the cell generates the signal similar to the mechanism described in Section 5.1. *delay* is the time between the release of *Messages* and the moment of their processing in the target gland releasing *Hormone*.

### 5.2 Propagation function: signaling via fiber/messaging via bloodstream

#### 5.2.1 Neuronal signaling

The fiber is the set of cells that offshoot with several compartments of axons or dendrites with the distributions of the following parameters:

1. *Fitness* values, similar to the nucleus sensitivity or concentration of receptors along the fiber, are used as the probability to generate a signal as a response to a hormonal message or neuronal signal.
2. *Signal transmission speed* or distribution of parameters, such as membrane conductance and capacitance, influences the speed of signal transmission along the offshoot membrane.

The level value *L* is formed by excitation/inhibition signals described in detail in Section 5.1. The fiber activation or membrane potential formation is similar to a nucleus with extension to the dynamics of the signal propagation:

$$L_{\mathrm{fiber}}\left(t\right)=\sum \left(L_{\mathrm{neuron}}\left(t-delay\right)-Decay\left(t-delay\right)\right),$$ (4)

where *delay* identifies the time from the signal formation in a neuron to the propagation to the particular destination site and *Decay* is the saturation function of the signal amplitude reduction; for a myelinated fiber, *Decay* = 0.

#### 5.2.2 Hormonal messaging

Hormonal messages consist of two types, as previously described in Renteln et al. (2011): (1) *suppressors* make nuclei and fibers that decrease the processing activity; (2) *accelerators* increase the processing triggered by a message. Message influence is represented in the following way: an output nucleus activity is formed by the *delay* in the hormonal messaging transport; thus, the relationship between triggering activity and produced hormones could be expressed using the following log–linear relationship formula: *Hormone*(*t*) = *e* ^−*Messages*(*t*−*delay*)^ (Hoermann and Midgley, 2012); for simplification purposes, we propose to use the power of 2:

$$\Delta Hormone\left(t\right)=2^{-Messages\left(t-delay\right)},$$ (5)

where *Messages* is the abstract amount of hormones released by a neuro-hormonal gateway, *delay* is inherited from Eq. 4, and Δ*Hormone* is an increase (acceleration)/decrease (suppression) in hormone production due to messages received.

## 6 Use cases

### 6.1 Hypothalamic–pituitary–thyroid axis

**FIGURE 7:**
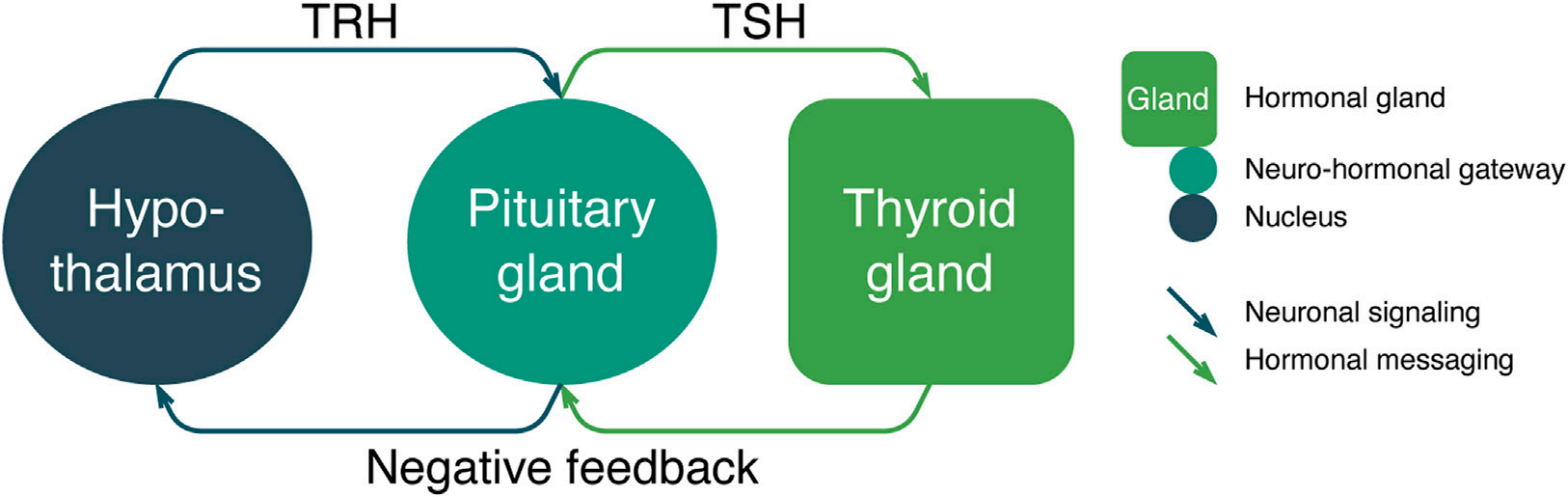
Hypothalamus–pituitary–thyroid axis (Sharlin, 2015) signaling/messaging and auto-regulation system, where hypothalamus, pituitary, and thyroid glands are groups of nodes and TRH, TSH, and negative feedback are hormonal messaging channels.

*Neuronal signaling and hormonal messaging*. The secretion of thyrotropin-releasing hormone (TRH) in the nucleus of the hypothalamus (group of nodes) (Figure 7) is activated via neuro-signaling. Later, the release of the thyroid-stimulating hormone (TSH) in the pituitary gland (group of nodes) which in turn, via the hormonal messaging, activates the thyroid gland (group of nodes) that produces thyroid hormones influencing the set of organs (groups of nodes). The thyroid gland has a negative feedback loop using hormonal messaging to the pituitary gland and neuronal signaling to the hypothalamus. The implementation of the proposed approach is as follows:

1. Hypothalamus neurons forming the TRH nucleus (group of nodes) start excitatory spiking (neuronal signaling) in the form described in Eq. 1 and thus form the output signal in the fiber from the hypothalamus to the pituitary gland (Eqs 2, 4).
2. The pituitary gland (group of nodes) receives the excitatory input (signal) and increases TSH production (Eq. 1).
3. Hormonal messaging with TSH transmits via the bloodstream, and later (taking into account delay) it reaches the thyroid gland (Eq. 3) and accelerates the production of thyroid hormones (Eq. 6).
4. The negative feedback is implemented via hormonal messaging, suppressing the production of TRH and TSH, thus auto-regulating the HPT system (Eqs 1, 6).

There are two types of propagation functions used in the example: neuronal from the hypothalamus to the pituitary gland and hormonal from the pituitary gland to the thyroid gland via the TSH. The messaging of TSH on the thyroid hormone is log–linear and could be implemented to save computational capacity as a power function of 2 (Eq. 5) (Geras and Gershengorn, 1982; Hoermann and Midgley, 2012) in the following way:

$$ThyroidHormone\left(t\right)=2^{-TSHMessages\left(t-delay\right)},$$ (6)

where *ThyroidHormone* is the level of thyroid hormone, *TSHMessages* is the amount of TSH released by the pituitary gland, and *delay* is the time between the release of *TSHMessages* and the moment of their processing in the thyroid gland.

### 6.2 Complete spinal cord injury

Advancements in artificial intelligence (AI) have led to the emergence of various novel techniques for analyzing pain levels and pain-related behaviors in gait analysis. One such innovative approach is hormonal computing in AI, which introduces a new paradigm to understand and classify pain-related data. This paper aims to provide a brief discussion of the differences between hormonal computing and conventional methods like deep learning in the context of pain level and pain-related behavior analysis in gait studies. To illustrate this comparison, we refer to a recent work, Dehshibi et al. (2023), where a GRU-based sparsely connected recurrent neural network (RNN) architecture was utilized for pain classification. In recent years, the study of pain and its implications in various fields has gained significant attention. Gait analysis is one area where understanding pain levels and pain-related behaviors is crucial for diagnosing and managing certain conditions. Traditional approaches to analyze pain data relied on deep learning techniques that have proven effective but possess limitations. Hormonal computing, as an emerging AI paradigm, offers an alternative method that could provide unique insights and potentially overcome some of the challenges faced by conventional techniques. The paper by Dehshibi et al. showcased the application of deep learning, specifically a gated recurrent unit (GRU)-based sparsely connected RNN architecture, for pain level and pain-related behavior classification in gait analysis. Deep learning has shown remarkable success in various AI tasks, but it often requires a substantial amount of labeled data, computational resources, and careful hyperparameter tuning. Furthermore, conventional deep learning models lack a direct connection to the underlying physiological processes that may influence pain perception. On the other hand, hormonal computing represents a new paradigm that draws inspiration from biological systems, specifically the endocrine system’s hormonal interactions. This approach leverages hormonal signals as a form of communication between AI agents. By integrating hormonal regulation into AI models, researchers aim to mimic the influence of hormones on decision-making processes and behavior in humans and animals. In the context of pain analysis, hormonal computing may offer a more biologically plausible framework for understanding pain perception and related behaviors. The key difference between hormonal computing and conventional deep learning lies in their underlying principles. While deep learning relies on large amounts of data for training and often acts as black-box models with limited interpretability, in contrast, hormonal computing introduces a bioinspired approach, allowing AI systems to communicate and regulate their behaviors in response to hormonal signals. Therefore, a major benefit of hormonal computing is its potential to incorporate domain knowledge and physiological insights directly into the AI model. By capturing the intricate interplay between hormones and pain perception, the hormonal computing approach may lead to improved accuracy and a deeper understanding of pain-related behaviors in gait analysis. In conclusion, the integration of hormonal computing in AI introduces a promising alternative to conventional deep learning methods for pain level and pain-related behavior analysis in gait studies. While conventional methods, such as deep learning models, have shown effectiveness in various applications, hormonal computing offers unique benefits by incorporating biological insights into the AI decision-making process. Future research in this area may provide further evidence of its utility and contribute to advancing pain analysis techniques in gait studies and beyond.

**FIGURE 8:**
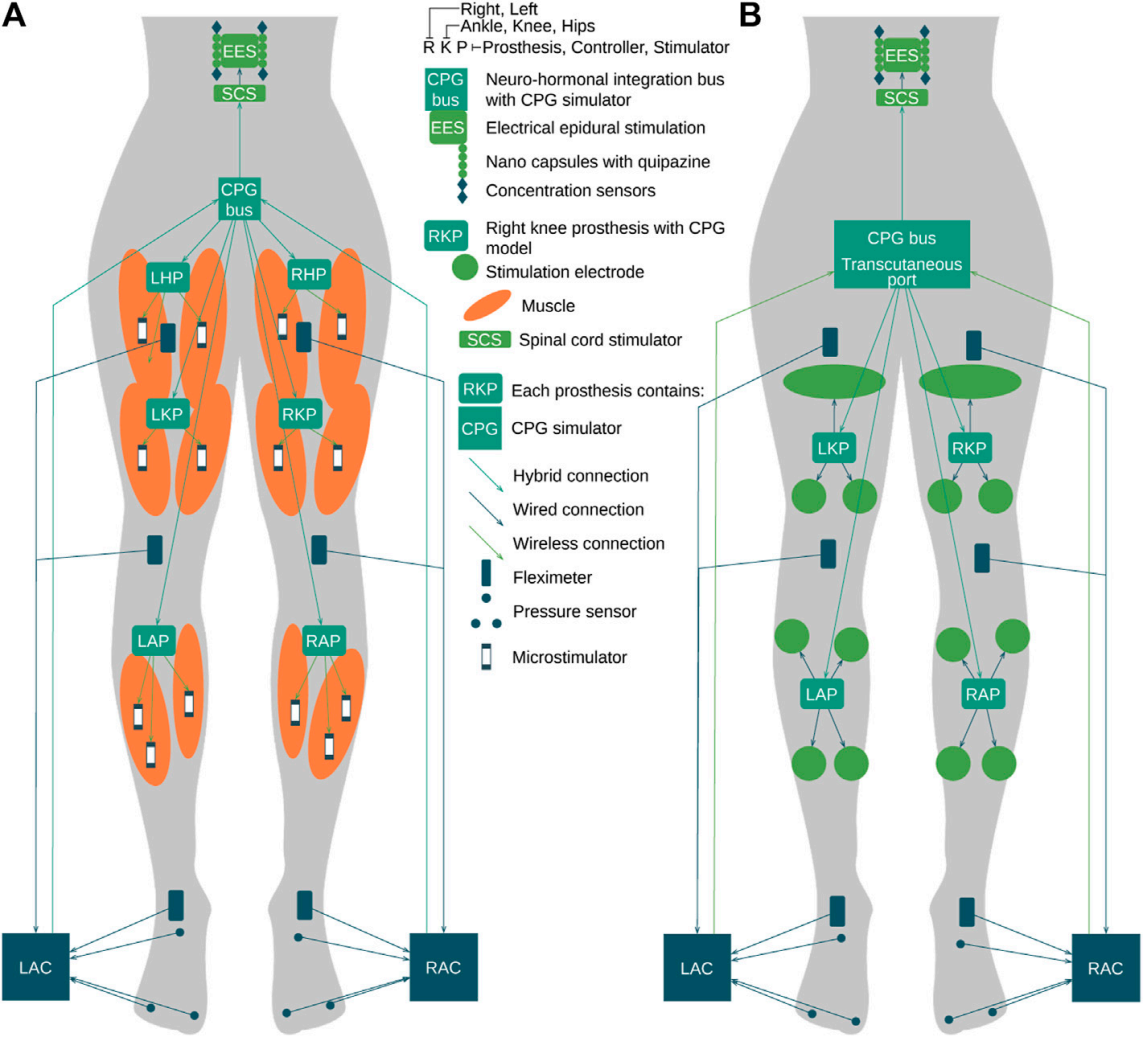
Example of CPG bus implementation. **(A)** Invasive implementation of the CPG neuro-hormonal message bus. **(B)** Hybrid architecture of the CPG neuro-hormonal message bus for the SC neurorehabilitation, where nanocapsules, EES, and the CPG bus are implemented invasively, while limb stimulators are connected to the bus via the transcutaneous port.

Currently, epidural electrical stimulation (EES) is widely used during complete spinal cord injury (SCI) neurorehabilitation (Wagner et al., 2018). Intraoperatively, the neurosurgeon identifies and adjusts the most effective combination of electrodes in the implanted array connected to the implant controller (Gill et al., 2018). In this work, we propose to extend current EES-based approach with the orchestrated stimulator infrastructure applied to limbs and spinal cord via neuro-hormonal prostheses: left hip prosthesis (LHP), right hip prosthesis (RHP), left knee prosthesis (LKP), right knee prosthesis (RKP), left ankle prosthesis (LAP), right ankle prosthesis (RAP), and chemical stimulator nanocapsules that release a cocktail of active substances in the area of a spinal cord (Figure 8). The orchestration of the aforementioned swarm of stimulators is performed via simulation of the spinal cord segment with a central pattern generator (implemented as CPG bus) that is influenced by sensors of neuronal and muscle electrical activity, force developed by muscles, and a concentration of active substances released by nanocapsules. The muscle stimulation is performed via a swarm of microstimulators synchronized in the hybrid manner described as follows (Loeb et al., 2006; DiLorenzo and Bronzino, 2008; Whitehurst et al., 2009; Becerra-Fajardo et al., 2017). The neuro-hormonal CPG integration bus implements the following cycle (Figure 8):

1. The release of the active substances (chemical hormonal message) is triggered by the “accelerate” message from the CPG bus.
2. Within *x* millisecond delay, stimulation of the spinal cord segment with the EES implant is triggered by the excitatory signals from the CPG bus.
3. Later, within *y* millisecond delay, the electrical stimulation of limbs with a walking pattern via swarm of the microstimulators is triggered by specific neuroprosthesis of the muscle group implemented via the set of oscillator motifs (Talanov et al., 2020), orchestrated in the CPG bus.

Here, *x* and *y* delays are identified by the neuronal model of CPG.

**FIGURE 9:**
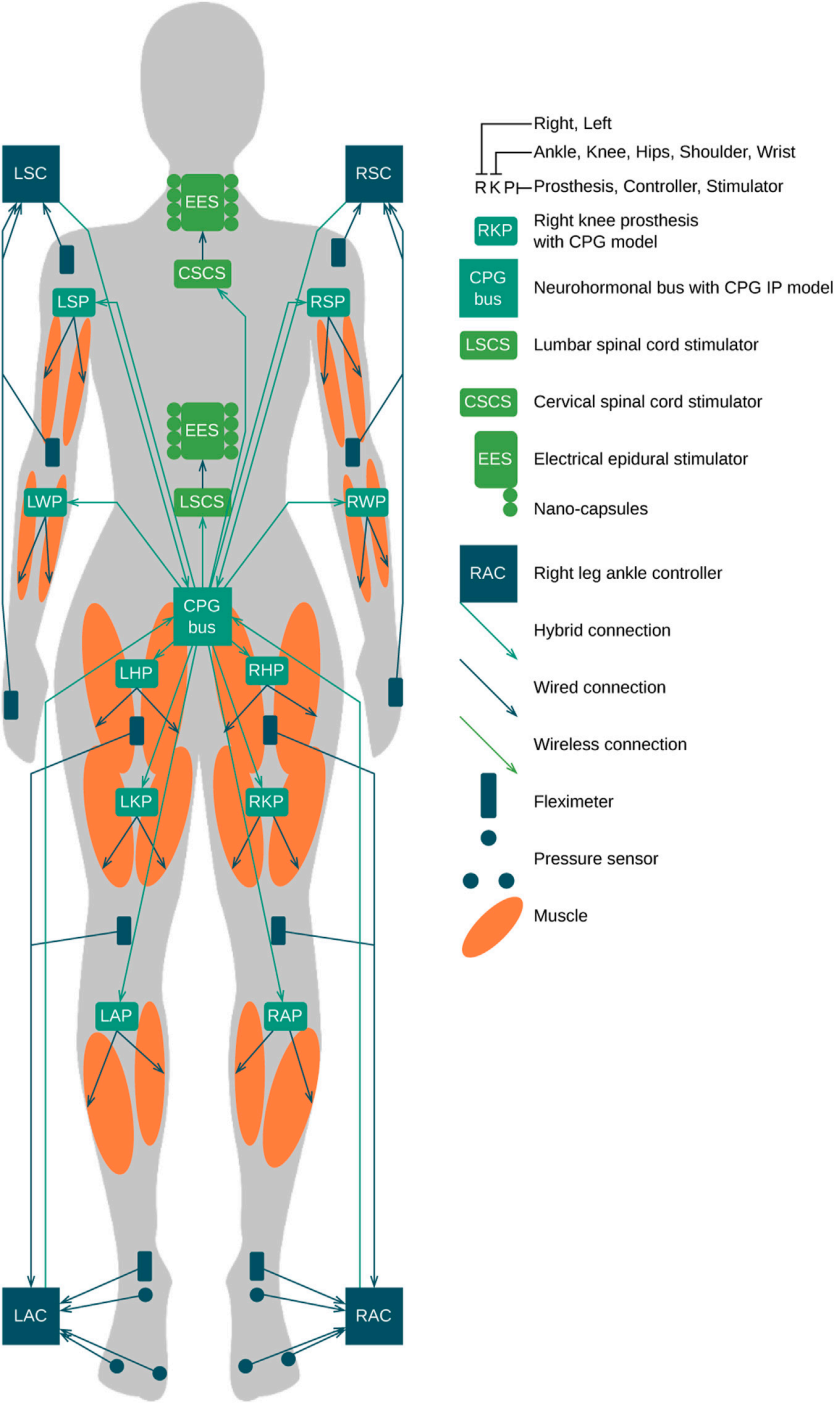
Complete SCI. High-level design of the hormonal orchestration architecture with neuromorphic neuroprosthesis with CPG bus infrastructure. Teal—CPG model bases computing machines and prostheses. Green—stimulators. Orange—muscles. Blue—input controllers, pressure sensors, and fleximeters.

The EES triggers the neuronal activity of the spinal cord segment to compensate for the lack of electrical activity from the brain. Active substances including quipazine facilitate the sensory input of the afferents (Gad et al., 2013) and accelerate the learning processes, enhancing the receptor trafficking to synapses (Zuzina et al., 2019; Zuzina and Balaban, 2022). During the influence of active substances, the stimulation of the limbs is generated in the walking pattern (Gad et al., 2013). Due to the EES and sequential muscle stimulation, the walking pattern circuitry in the spinal cord is built due to self-organization (Gill et al., 2018; Wagner et al., 2018). The concentration of the quipazine must be tracked to account for the quipazine degradation by specialized sensors to generate proper stimulation of limbs. The combination of the EES, quipazine stimulation, muscle stimulation, and physical exercise provides the most effective influence on the patient’s spinal cord and muscles and limbs. We propose two implementation options: (1) the invasive option where microstimulators are implanted in the muscle tissue as well as prostheses implanted near stimulators to guarantee stable connection (Figure 9); (2) the non-invasive option implies the hybrid implementation of prostheses as implants and external stimulators managing muscles using transcutaneous electrical impulses of the muscle stimulation (Figure 9).

The implementation and extension of the proposed approach are applied in the case of a high complete SCI C1 model (Figure 8). (1) The hormonal message bus triggers the EES above (CSCS) and below (LSCS) the trauma. (2) The prosthesis swarm has a specific CPG model for each limb mounted in hands: LWP, LSP, RWP, and LWP and in legs: LAP, LKP, LHP, RAP, RKP, and RHP and has their input controllers in hands: LSC and RSC and in legs: LAC and RAC connected to pressure sensors and fleximeters monitoring the angles of ankles, knees, hips, wrists, elbows, and shoulders. (3) Implanted microstimulators are orchestrated through the hybrid connection by prostheses listed previously with the specific muscle group of the CPG model implemented and executed in real time. Because the Bluetooth signal could transmit through approximately 80 mm of muscle tissue (Christoe et al., 2021), microstimulators should be placed in stimulated muscle and receive synchronization signals wirelessly from the model-based prosthesis, while antennas should be placed directly under the skin. The other approach could be the use of ultrasound for the connectivity between prostheses and stimulators. Each prosthesis is managed by the CPG bus that contains the complete CPG model responsible for the synchronization of limbs and flexor–extensor muscles.

**FIGURE 10:**
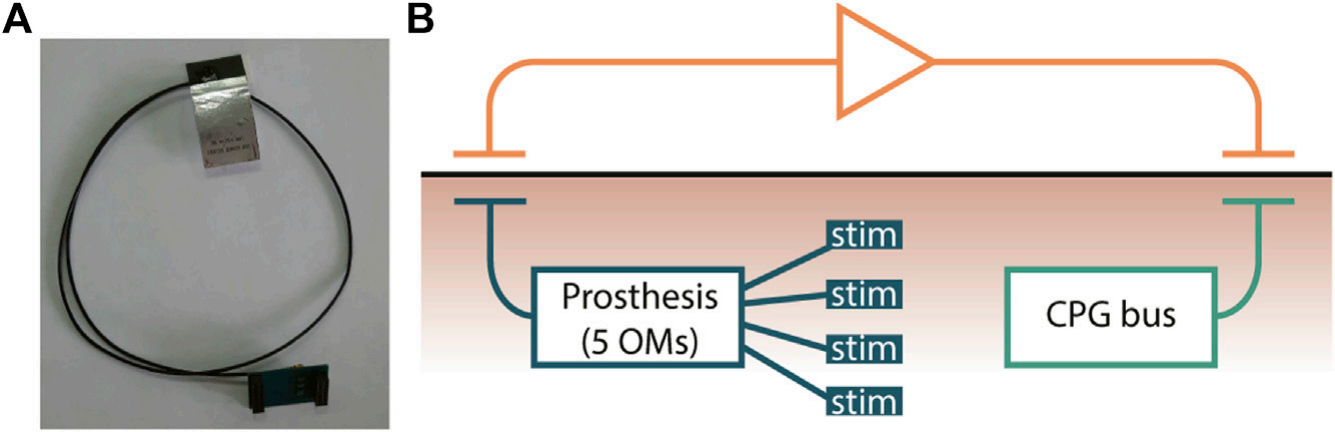
Bluetooth flat antenna of the transcutaneous connections. **(A)** Typical Bluetooth flat antenna with UMCC (Ultra-Miniature Coax Connector). **(B)** Hybrid connection schematic where the prosthesis with five OMs manages microstimulators implanted in the muscle tissue via the Bluetooth transmitting antenna under the skin and the receiving antenna is attached to the skin, which is connected to an amplifier and later to a transmitting antenna near the prosthesis receiving antenna.

The Bluetooth flat antenna for transcutaneous connectivity (Figure 10A) is attached to the skin near the prostheses and forms a hybrid connection with the prosthesis (Figure 10B). The implantable CPG bus hybrid connection is organized from each prosthesis in the legs and hands to the bus via two-ended antennas, and the input controllers are connected with the CPG bus via a Bluetooth antenna mounted near the implanted bus. The advantages of this approach are as follows: (1) allowing a patient to be more mobile; (2) not depending on clothes and protected by the skin; and (3) because of the use of two-ended antennas, there is no need to use Ultra-Miniature Coax Connector (UMCC); the disadvantages of this approach are as follows: (1) antennas of transcutaneous connection must be located over the implanted devices for stable wireless connection. The non-invasive implementation contains a hybrid connection using one-ended antennas to each prosthesis and UMCC connection with the CPG neuro-hormonal bus, with the following advantages: (1) easy installation, (2) no surgery required, (3) less Bluetooth power consumption, and (4) the size of the bus could also be increased for extended functionality; and disadvantages: (1) if the bus is not attached to the body, the whole system is not functional and (2) the implementation could be used only indoors.

We proposed the orchestrated architecture of swarms of implants mounted in muscle tissue or near nerves to manage limbs as the implementation of the hormonal computing and neuro-signaling and hormonal messaging approaches. The implementation could be quite flexible in terms of the implantation of stimulators, prostheses, and neuro-hormonal buses; they could be implemented in invasive and non-invasive ways. Both ways have their limitations: invasive requires surgery but allows for stimulation of deep muscles and high subject mobility, whereas non-invasive requires no surgery but could be used only indoors for safety reasons from environmental precipitation and electromagnetic noise.

## 7 Delivery and release of hormonal substances: possible hardware implementations

When the necessity of hormone support is identified, important questions arise: how to deliver them to the proper places of the body and to release them in an adequate moment?

It is possible to distinguish two situations: when the place is well defined (in the case of prosthesis or implants) and when its location can be varied, but trackable. Let us consider these two cases separately.

### 7.1 Defined place

**FIGURE 11:**
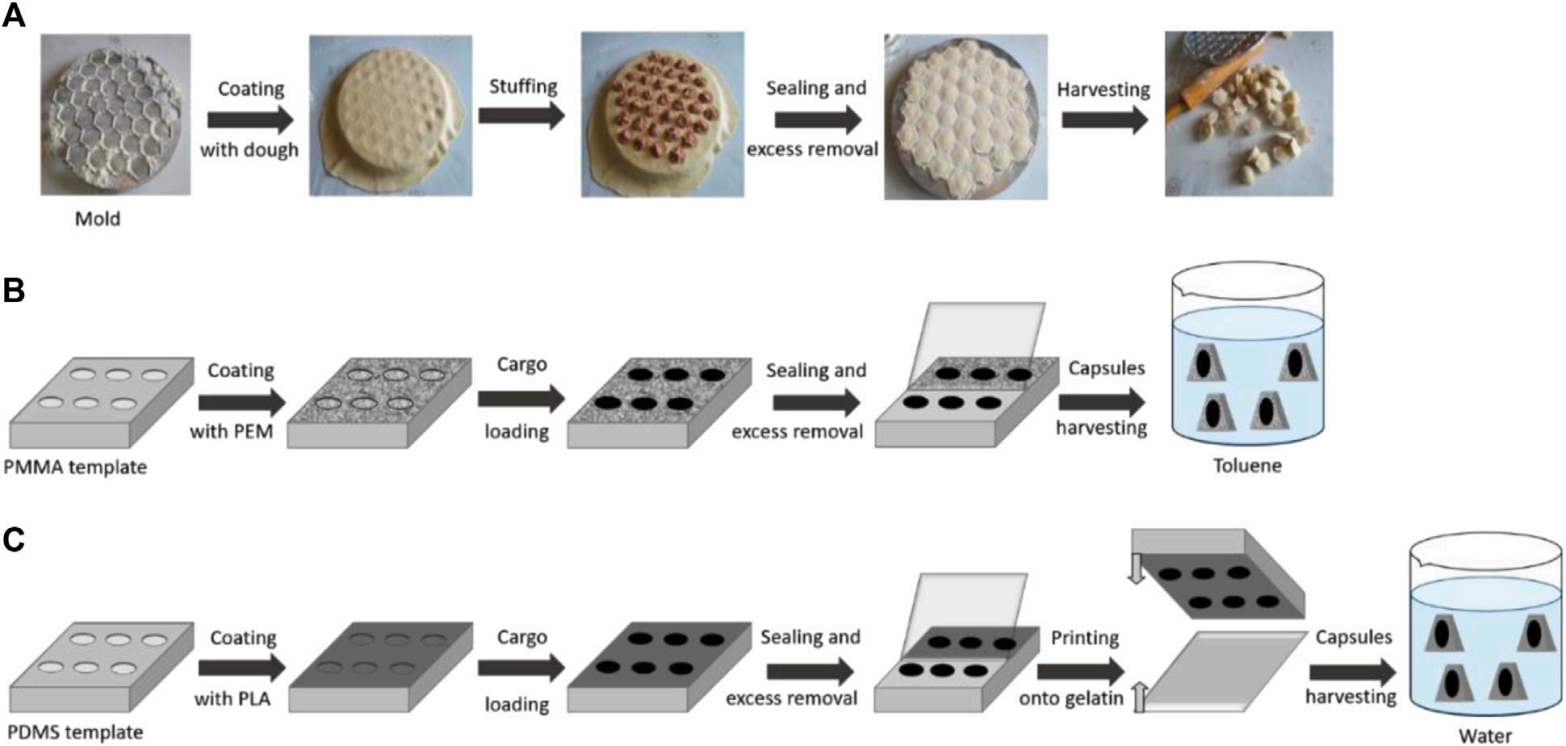
Schematic illustration of defined-shape microcapsule fabrication. **(A)** Traditional pelmeni production process; **(B)** PEM microcapsule harvesting in toluene; and **(C)** PLA microcapsule harvesting in water (Kudryavtseva et al., 2021)

In the case when the position of the possible release of these compounds is well defined, as in the case of prostheses or implants, the strategy can be based on the immobilization of smart microchambers on their surfaces. The technique has been known since 2011 (Kiryukhin et al., 2011), and it allows the fabrication of micron-size containers, filled with necessary pharmaceutical preparations, on the surface of solid supports. Several approaches are used for the realization of these chambers. However, discussing these approaches are out of scope of this study and can be found in Antipina et al. (2014), Gai et al. (2016), and Kudryavtseva et al. (2021). The approach is schematically illustrated in Figure 11.

It is important to mention only that the surface of the prosthesis/implant can contain chambers, filled by the desirable compounds (hormones, in this case). Therefore, the implant/prosthesis will already have containers, filled with the pharmaceutical preparations, that will be, very likely, useful in the future function of these items. Thus, the place of the localization of the drug sources is defined, and it is necessary only to trigger their release in the adequate moment.

In the past, opening of these chambers was performed one after another by irradiation using laser light, showing very high efficiency. In the case of simultaneous opening of all chambers, it is possible to use ultrasound or alternating magnetic fields. However, the simultaneous release of all encapsulated material is not suitable for the purpose of this paper, as it will work only once and then the implant/prosthesis should be substituted, increasing the cost of the operation and decreasing the wellness of the patient. Thus, these chambers must be opened one after another, allowing dosed release of hormones, when it is necessary for the organism.

As it was mentioned previously, one-by-one openings can be effectively performed by laser illumination. However, this approach will demand the use of optical fiber, penetrating into the body, adequate scanning system, and feedback, monitoring the actual concentration of the drug (hormone), and comparing it with the required one. Thus, it will demand the attachment of rather heavy and complicated equipment that the patient will need to have her/his body attached to equipment, which will not, of course, improve the quality of life.

Instead, recently, the principle possibility of opening chambers and inducing release by the application of adequate electrical pulses was demonstrated (Ricci, 2020). The suitable configuration of electrodes will allow the opening of chambers row by row, column after column, and even chamber after chamber. Thus, no scanning and insertable instruments are required. The only remaining questions are how to identify the necessary concentration of drugs, triggering only the required number of chambers to open, and how to realize the electronic circuit, avoiding direct electrical connections with wires to the prosthesis/implants.

In order to suggest how to perform it, let us consider a chip developed in EPSL (Baj-Rossi et al., 2014). The chip was designed and realized as a wireless sensoristic system. Originally, it was a 1 cm × 1 cm implantable chip, 80% of its area was a planar helical antenna, used for the power supply of all electronics in the chip. The remaining 20% of the area was used for temperature and pH measurements, along with four enzymatic sensors. Current versions of similar devices have even significantly reduced sizes.

However, the basic principle can be based on the original idea (Baj-Rossi et al., 2014). In the case of a prosthesis/implant, we can realize a planar antenna on its surface. In addition, the item will contain chambers with hormones, a system of electrodes to trigger the opening of these chambers and release encapsulated hormones, and, optionally, sensors, controlling the actual concentration of hormones and comparing it with the desirable one. In the last case, the system must include sensor elements.

Thus, the immobilized sensor system determines the necessary amount of the drug (hormone) to be released and sends the signal, by means of the planar antenna, to the central station (personal computer or smartphone), triggering the opening of the required number of chambers for releasing the necessary amount of the encapsulated hormones.

### 7.2 Circulating carriers: varied but trackable location

The other case requires a continuous supply of pharmaceutical preparations (hormones), when the targeting area is only roughly determined and the release time can also be variable. Thus, carriers must have the possibility of moving, delivering hormones to a desirable area, and waiting for the release time. In this respect, it is interesting to consider nanoengineered polymeric capsules.

Here, we will not consider the technical details, just underlining important steps of the preparation and properties of these objects.

Polyelectrolyte self-assembly, also known as the layer-by-layer (LbL) technique, was used for the first time for the realization of polymeric structures by Decher (1997). The technique became very popular straight away as it does not require special equipment. Nevertheless, it allows to fabricate structures with nanometer resolution (in one direction). Thus, the technique became very popular among groups working in the field of molecular systems with nm resolution.

The next important step was performed by Sukhorukov et al. (1998): the method can be applied not only to planar systems but also to spherical (or any other shape) objects. It was necessary to separate templates with already deposited layers from the reacting solution and wash them carefully and to expose to solutions of oppositely charged objects. Nevertheless, these objects have immediately attracted the attention of numerous research groups, mainly due to the fact that the assembling can be performed on sacrificial templates that can be dissolved after the realization of a desirable sequence of layers in the shell. Thus, it is possible to make empty objects with micron or sub-micron sizes, with shells containing layers of functional molecules with nm resolution. The internal part of these objects can be filled by active molecules (hormones, in our case).

Summarizing, these objects can be considered ideal smart containers for drug delivery and release (Pastorino et al., 2013) and as prospective candidates for biochemical unconventional computing (Erokhina et al., 2015).

In fact, the internal part of these capsules can be loaded with active compounds, while the shell architecture will allow directed delivery (magnetic nanoparticles for the rough delivery; receptors and/or antibodies for the fine delivery) and induce release (pH and/or ionic strength variation; UV, visible, IR, or microwave irradiation; and ultrasonic)

In the case of pharmaceutical applications, the ideal container must provide the possibility to deliver the therapeutic preparation to the disease risk zone and to release it when it is required. In the case of the use in the field of unconventional biochemical computing, the loaded internal part of capsules can be considered “a main executive program,” while the shell architecture serves as a “demon listening program,” whose task is to deliver the “main program” to the required area and to analyze the environmental conditions, being ready to start it when necessary (Erokhina et al., 2015).

Thus, if the place of the delivery and release is not defined, hormone-containing carriers can be injected into the blood and delivered to the defined place by, for example, an external magnetic field. Ideally, the release must be triggered automatically when it is required. In this case, the shell architecture must be realized in such a way that pores will be open only when the environment will require additional hormones (Pastorino et al., 2013). However, it is not always feasible. Nevertheless, if the containers are delivered to defined zones and implanted sensors will register the necessity of the release of the hormones, triggering can be performed remotely, using UV, visible, or IR illumination and ultrasound or microwaves.

Summarizing, the current state of the art allows the effective delivery and release of hormones to required zones of the human body, which can be controlled by the internal activity of the body and/or by signals from external computers.

## 8 Ethical concerns

Hormonal computing is an emerging field that explores the incorporation of hormone-based mechanisms into AI, neuroengineering, and computational systems. This technology holds significant promise for enhancing the capabilities of these systems, but it also raises significant ethical concerns that need to be carefully addressed. Here, we analyze the following problems:

1. Informed consent and autonomy: As hormonal computing delves into neuroengineering, there may be ethical challenges related to informed consent and individual autonomy. When using hormonal interventions to influence cognitive functions or emotions, the boundary between enhancing capabilities and potentially manipulating an individual’s thoughts and feelings becomes blurred. It is crucial to ensure that users are fully informed about the potential effects of hormonal interventions and have the right to make informed decisions about their involvement.
2. Privacy and data security: Hormonal computing involves the collection and analysis of highly personal and sensitive biological data related to hormone levels and responses. Ensuring the privacy and security of this data becomes paramount, as any breaches could lead to serious implications for individuals’ wellbeing and could potentially be exploited for nefarious purposes.
3. Bias and fairness: Incorporating hormonal computing in AI systems could introduce biases based on hormonal differences among various populations. These biases may perpetuate existing societal inequalities, especially if they influence decision-making processes, such as in hiring or medical diagnoses. Ethical consideration should be given to mitigating these biases and ensuring fairness and equal treatment for all individuals.
4. Unintended consequences: The complex nature of hormonal systems makes the prediction of the full extent of outcomes challenging. Introducing hormonal computing into AI and computational systems might lead to unintended consequences at both the individual and societal levels. Researchers and developers must thoroughly assess and anticipate potential risks to prevent any adverse effects on users and society as a whole.
5. Manipulation and control: The ability to influence emotions and cognitive functions through hormonal computing raises concerns about potential misuse. If this technology falls into the wrong hands, it could be utilized for manipulative purposes, such as altering behavior or inducing specific emotional states without the user’s consent.
6. Medical and psychological implications: Application of hormonal computing in neuroengineering could have medical and psychological ramifications. The technology might offer new treatments for certain conditions, but it also raises questions about medical oversight, potential side effects, and long-term impacts on an individual’s wellbeing.

Mitigating the ethical concerns related to hormonal computing in AI, neuroengineering, and computational systems requires a combination of measures to ensure responsible development and use of this technology. Some strategies to address these concerns are as follows:

1. Informed consent and autonomy:
  a. Implement clear and transparent informed consent processes, providing users with comprehensive information about the purpose, risks, and potential benefits of hormonal interventions.
  b. Empower individuals to make autonomous decisions about their participation in hormonal computing systems without coercion or undue influence.
  c. Establish clear guidelines for withdrawing consent and ensure that users have control over their data and the use of hormonal interventions.
2. Privacy and data security:
  a. Adhere to strict data protection and privacy regulations, ensuring that sensitive biological data collected for hormonal computing is securely stored, transmitted, and accessible only to authorized personnel.
  b. Implement robust encryption and anonymization technologies to protect individual identities and prevent data breaches.
3. Bias and fairness:
  a. Conduct thorough audits and bias assessments of AI and computational systems that incorporate hormonal computing to identify and address potential biases.
  b. Promote diversity and inclusivity in the development and training of these systems to minimize bias in decision-making processes.
  c. Regularly update and improve algorithms to reduce biased outcomes and ensure fairness in the application of technology.
4. Unintended consequences:
  a. Prioritize extensive testing and simulations to anticipate and identify potential unintended consequences of hormonal computing.
  b. Establish continuous monitoring and feedback mechanisms to detect any adverse effects or unexpected outcomes promptly.
  c. Collaborate with multidisciplinary teams and subject matter experts to evaluate the technology’s implications thoroughly.
5. Manipulation and control:
  a. Establish strict regulations and ethical guidelines for the use of hormonal computing to prevent misuse and manipulation.
  b. Conduct independent audits and oversight of applications using hormonal computing to ensure compliance with ethical standards.
  c. Promote transparency in the use of hormonal computing technology and its intended purposes.
6. Medical and psychological implications:
  a. Involve medical professionals and psychologists in the development and implementation of hormonal computing systems to assess potential risks and benefits.
  b. Conduct extensive clinical trials and studies to validate the safety and efficacy of hormonal interventions used in neuroengineering and medical applications.
  c. Ensure that the application of hormonal computing in medical contexts adheres to established ethical guidelines and principles.
7. Public awareness and education:
  a. Raise awareness among the public, policymakers, and stakeholders about the ethical concerns and implications of hormonal computing.
  b. Promote public discussions and engagement to gather diverse perspectives and ensure that ethical considerations shape the development and regulation of this technology.

By adopting these strategies and promoting a culture of responsible innovation, we can mitigate the ethical concerns surrounding hormonal computing, fostering its safe and beneficial integration into AI, neuroengineering, and computational systems.

## 9 Conclusion

This paper has explored two existing ways of using bioinspired hormonal models to provide some engineering responses to real-time problems, as well as two different novel ways to create computational resources for the possible implementation. With all these examples, we have shown the multiple benefits of the use of bioinspired hormonal models for the sake of solving existing engineering and computational challenges. The dynamic, decentralized, and multifunctional properties of the hormonal systems allow them the creation of innovative and complex systems.

## Data Availability

The original contributions presented in the study are included in the article/Supplementary Material; further inquiries can be directed to the corresponding author.
